# Alterations in Human Hippocampus Subregions across the Lifespan: Reflections on White Matter Structure and Functional Connectivity

**DOI:** 10.1155/2023/7948140

**Published:** 2023-03-28

**Authors:** Jianling Tan, Zhongyan Wang, Yi Tang, Yin Tian

**Affiliations:** Department of Biomedical Engineering, School of Bioinformatics, Chongqing University of Posts and Telecommunications, Chongqing 400065, China

## Abstract

During growth and aging, the role of the hippocampus in memory depends on its interactions with related brain regions. Particularly, two subregions, anterior hippocampus (aHipp) and posterior hippocampus (pHipp), play different and critical roles in memory processing. However, age-related changes of hippocampus subregions on structure and function are still unclear. Here, we investigated age-related structural and functional characteristics of 106 participants (7-85 years old) in resting state based on fractional anisotropy (FA) and functional connectivity (FC) in aHipp and pHipp in the lifespan. The correlation between FA and FC was also explored to identify the coupling. Furthermore, the Wechsler Abbreviated Scale of Intelligence (WASI) was used to explore the relationship between cognitive ability and hippocampal changes. Results showed that there was functional separation and integration in aHipp and pHipp, and the number of functional connections in pHipp was more than that in aHipp across the lifespan. The age-related FC changes showed four different trends (U-shaped/inverted U-shaped/linear upward/linear downward). And around the age of 40 was a critical period for transformation. Then, FA analyses indicated that all effects of age on the hippocampal structures were nonlinear, and the white matter integrity of pHipp was higher than that of aHipp. In the functional-structural coupling, we found that the age-related FA of the right aHipp (aHipp.R) was negatively related to the FC. Finally, through the WASI, we found that the age-related FA of the left aHipp (aHipp.L) was positively correlated with verbal IQ (VERB) and vocabulary comprehension (VOCAB.T), the FA of aHipp.R was only positively correlated with VERB, and the FA of the left pHipp (pHipp.L) was only positively correlated with VOCAB.T. These FC and FA results supported that age-related normal memory changes were closely related to the hippocampus subregions. We also provided empirical evidence that memory ability was altered with the hippocampus, and its efficiency tended to decline after age 40.

## 1. Introduction

The hippocampus is one of the key areas in cognitive processes such as learning, memory, and future imagination across the lifespan (from development to ageing), which is particularly vulnerable to aging [1, 2]. The hippocampus is divided into anterior hippocampus (aHipp) and posterior hippocampus (pHipp) along the hippocampal axis [3]. Back in the 1960s, Nadel was keenly aware that the dorsal (posterior in human) and ventral (anterior in human) hippocampi likely facilitate different functions [4]. But at that time, he did not derive a full explanation for the discrepancy. Many people have gone on to notice this anterior-posterior distinction adding further to the phenomenon.

The dorsal hippocampus in primates is more associated with spatial processing compared to the ventral [5]. Both the anterior and posterior hippocampi show age-related structural volume reduction, but the structure and function of the aHipp may be susceptible to aging [6]. During memory encoding and retrieval, the aHipp and pHipp interact with regions of the attention network and the default network, respectively [7]. The aHipp and pHipp regions form functionally distinct “circuits” and have distinct anatomical connections to other brain regions. For example, the anterior hippocampus (head and body) is preferentially functionally connected to the default mode network, and the hippocampal tail is strongly preferentially functionally connected to the parietal memory network [8, 9]. With the increasing wealth of such findings, there is a great interest in the functional and structural specificity of different neural groups between aHipp and pHipp. Meanwhile, an interest in the impact of lifespan development on the aHipp and pHipp also increases. So far, researchers have provided different and interesting findings on hippocampus.

In regard to functional connectivity, the function of the hippocampus and its adjacent cortices have been found to show age invariance in some cases [10], but age-related increase [11] or decrease in others [12]. Salami et al. explored the relationship between FC of hippocampus and memory deficits in lifespan; an age-related U-shaped trajectory of FC was found [13]. Langnes et al. found that strong FC within the hippocampus restricted the degree to which the hippocampus interacted with other brain regions [14]. Given the functional distinctions between hippocampus subregions, it was likely that the anterior and posterior hippocampi facilitated different functions through their differential connections with the cortex. For example, functional studies have investigated changes in FC of the hippocampus and networks of relevant brain structures from childhood to adulthood. They found that aHipp and pHipp supported memory formation and identified age differences in memory-related differential hippocampal FC with several frontal and visual/sensory cortices [15, 16]. Blum et al. reported that the FC in the pHipp was more dominant with age, which supported age-related reorganization of the hippocampus network for normal cognitive function [17]. In addition, the study from Stark et al. further showed that hippocampal circuit alterations which was affected by hippocampal subfields were associated with age-related memory decline [18].

There are some inconsistencies in the results obtained in these available literatures. The reason for these may lie, and they only explored the relationship between the hippocampus and a single functional area, so ignore the interactive effects of the multifunctional area on the hippocampus. However, the analysis from single brain region to brain connectivity, reported recently in science, showed that the key to the realization of a certain function was not the independent completion of each brain region but the connection and communication between multiple regions [19]. The cognitive capacity (intelligence) of the human brain may be more reflected in the connectivity of multiple regions.

Hippocampal structure has different developmental trajectories for young and older individuals. Riggins et al. reported the influence of age on hippocampal development and its relations with memory ability earlier in life. Results examining hippocampus subregions (i.e., subiculum, CA1, and CA2-4/DG) suggested smaller CA1 and larger CA2-4/DG contributed to better memory performance [20]. Eguchi et al. explored the relationship between cognitive function and hippocampal structure in the elderly (≥95 years), and regression analysis found a significant relationship between ACE-III memory scores and hippocampal subregion volume [21]. In agreement with previous studies, Pereira et al.'s results showed that age had a significant negative effect on volume in CA2-4/DG [22]. Importantly, they also found a negative age effect on FA in the subiculum. Amenta et al. investigated astrocyte changes in aging hippocampus [23]. They found that an increase in the number and size of astrocytes was observed in the CA1 with age. Dalton et al. analyzed the patterns of hippocampus subregions FC in the context of healthy ageing [24]. Compared to the young group, they found that the older participants had significantly reduced FC between the CA1-subiculum and the entorhinal cortex.

Diffusion tensor imaging (DTI) was very sensitive to changes in microstructural white matter [25]. Fractional anisotropy (FA) characterized the directionality of constrained water diffusion in the brain tissue [26]. FA values might reflect how efficiently information was transmitted and thus be related to the functional response to a stimulus [27, 28]. Hence, it was commonly employed to explore the structural integrity of tissue microstructure on white matter. For example, the researchers used FA to examine relationship between hippocampal white matter structure and age [29, 30]. Sander et al. have shown that hippocampal structural integrity was affected by aging, resulting in lower success in memory encoding in older adults compared to younger adults [30]. Although a large number of structural and functional imaging studies have reported altered structural and FC in hippocampus, only a few studies on patient with epilepsy have directly investigated the interplay between structural and FC in hippocampus [31, 32]. Structure was generally considered the substrate for FC [33], but no consensus has been reached on a relationship between structure and function across lifespan [34, 35].

Therefore, our study is aimed at exploring age-related changes in hippocampal function and structure in a more comprehensive way. We also focused on the hippocampus subregions and the role of multiple functional brain regions to gain insight into the neural basis of the hippocampus in the process of human brain development and aging. Based on the previous findings, we predict that with normal aging, functionally, changes in hippocampal FC strength are limited by the function of other brain regions. Structurally, hippocampal white matter integrity is progressively diminished and strongly correlated with cognitive performance. To test these hypotheses, we explored the changes in FC of aHipp and pHipp within the hemispheres. Meanwhile, we combined with white matter structural integrity (based on FA) of aHipp and pHipp to obtain age-related changes. These would help us to better understand the separation and integration in structure and function of the memory-related divisions across the lifespan. Thus, this study had scientific implications for understanding the changes in hippocampal structure and function with age. The trajectory of the hippocampus with age could provide evidence for theories of cognitive development.

## 2. Materials and Methods

The dataset was obtained from the Nathan Kline Institute/Rockland Sample (NKI–RS1: http://fcon_1000.projects.nitrc.org/indi/pro/nki.html). Here, the sections of data acquisition, fMRI data preprocessing, DTI data preprocessing, and age-related generalized linear model were similar to our previous article methods [28, 36].

### 2.1. Participants

The sample consisted of 106 participants (46 females, age range 10-85 years, mean = 38.8 years; 60 males, age range 7-81 years, mean = 38.3 years; age ≤ 15, *n* = 11; age 16-25, *n* = 32; age 26-45, *n* = 24; age 46-65, *n* = 23; and age ≥ 65, *n* = 16). All participants are right-handed and not diagnosed with any mental abnormalities. All data acquisition and sharing were approved by the institutional review board of the Nathan Kline Institute. All participants provided written informed consent. For child participants who were unable to provide informed consent, written informed consent was obtained from their legal guardians.

### 2.2. Data Acquisition

All initial fMRI and DTI data were obtained with a 3T Siemens Trio scanner. High-resolution T1-weighted structural data were obtained with magnetization-prepared rapid gradient echo sequence (TR = 2,500 ms, TE = 3.5 ms, flip angle = 80°, thickness = 1.0 mm, slices = 192, in‐plane matrix = 256 × 256, and field of view = 256 mm). T2-weighted functional data were obtained with a single-shot, gradient-recalled echo-planar sequence (TR = 2,500 ms, TE = 30 ms, flip angle = 80°, field of view = 216 mm, in‐plane matrix = 64 × 64, slices = 38, thickness = 3.0 mm, and volumes = 260). DTI was conducted with sequence parameters (TR = 10,000 ms, TE = 91 ms, field of view = 256 mm, and slices = 58), non-diffusion-weighted images (*b* = 0 s/mm^2^), and 64-direction diffusion-weighted images (*b* = 1,000 s/mm^2^). In order to ensure steady-state longitudinal magnetization, the first 4 volumes were excluded.

### 2.3. fMRI Data Processing

#### 2.3.1. Preprocessing

A sequence of steps was applied to preprocess the dataset using the Statistical Parametric Mapping software based on SPM12 (http://www.fil.ion.ucl.ac.uk) and REST software (http://restfmri.net/forum/REST_V1.8). This procedure involved the following: (1) slice-timing correction was performed to correct each voxel's time series during acquisition. (2) Functional images were realigned for head-motion correction with rigid-body transformation (translation ≤ 1.5 mm and rotation ≤ 1.5°: no participant's head motion exceeded these values when looking across their whole scan). (3) Images of each subject are registered to the individual subjects' T1 structural image, and then, data were spatially normalized to the Montreal Neurological Institute (MNI) standard template, and voxels resampled to 3 × 3 × 3 mm^3^. (4) Data were spatial smoothed using a Gaussian kernel with a 5 mm full width at half maximum (FWHM) to ensure a high signal-to-noise ratio (SNR). (5) To remove the linear low frequency drift and physiological noise, low pass filtering was performed to extract the low frequency signal range over 0.01-0.10 Hz. (6) Considering the impact of microlevel head motion on FC, we further corrected for head motion more finely by excluding 24 sources of variance via a Friston 24-parameter model (a model that can remove movement-related artifacts from fMRI time series, quickly, automatically, and with a degree of validity) [37], including 6 parameters derived from the rigid-body head motion correction, 6 parameters of head motion one time point before, and 12 corresponding squared items, and we also removed the mean signals over the whole brain, white matter, and cerebrospinal fluid. The empirical analyses suggested that over 90% of fMRl signal can be attributed to movement and that this artifactual component can be successfully remove [37]. In the subsequent statistical analysis, we eliminated the head motion as a covariate.

#### 2.3.2. Identifying Hippocampus Subregions for Regions of Interests (ROIs)

In order to explore the age-related memory alterations in human hippocampus subregions, four brain ROIs in hippocampus were selected with MNI coordinates. For aHipp, bilateral coordinates were selected (right aHipp (aHipp.R): 18, -14, and -18; left aHipp (aHipp.L): -18, -14, and -18) [13]. For pHipp, the bilateral coordinates were selected (right pHipp (pHipp.R): 25, -26, and -15; left pHipp (pHipp.L): -23, -26, and -15) [38]. The average time series of the spherical seed ROI with a radius of 4 mm were extracted. The cortical brain areas were parcellated by anatomical automatic labeling (AAL) from Montreal Neurological Institute. The same parcellation scheme was used across all participants.

#### 2.3.3. Acquiring FC for ROIs after Preprocessing fMRI Images

For the calculation of FC within the same hemisphere, we computed a Pearson's product moment correlation coefficient, *r*, between the averaged time series within each seed region and the time series of all voxels in its cerebral hemisphere (AAL). Next, the resultant correlation maps were converted to *z* (*r*) values using Fisher's *r*-to-*z* transformation. Statistical analysis was performed for the Fisher-*z* transformed FC.

### 2.4. DTI Data Processing

#### 2.4.1. Preprocessing

DTI data were preprocessed using a sequence of standard steps based on voxel-based analysis (VBA), and processing was conducted with the SPM8. This procedure involved the following: (1) correction for eddy-current-induced distortion and head movement using affine registration to the imaging volume (*b* = 0) with no diffusion weighting; (2) evaluating diffusion tensor metrics and FA on each voxel; (3) registering all the individual FA images to the FA template in the MNI space; and (4) spatial smoothing using a 6 mm full width at half maximum Gaussian kernel for high signal-to-noise ratio and resampling to 2 × 2 × 2 mm^3^ voxels.

#### 2.4.2. Extracting FA after the Preprocessing DTI Images

We extracted the mean FA values for each constructed hippocampus ROI (same as described above) from the segmented map. Then, the Pearson correlation coefficient (*p* < 0.05) between FA values and FC values was calculated as mentioned above for each hemispheric hippocampus subregion (*p* < 0.05, with voxel-wise false discovery rate (FDR) corrected) to explore the relationship between hippocampal function and structure.

### 2.5. Statistical Analysis

#### 2.5.1. One-Sample *t*-Test and Paired *t*-Test

The signal-to-noise ratio was equivalent for aHipp or pHipp in all analyses. Following current reporting standards, all statistical results were corrected for familywise error (FWE) of multiple comparisons based on the Gaussian random field theory, as implemented in the SPM.

*(1) FC Values*. Within-group analysis of Fisher-*z* transformed FC from hippocampus subregions was conducted using one-sample *t*-test, by which we compared the *z*-value of each voxel to a normal distribution with mean of zero and an unknown variance (*p* < 0.05, FWE corrected). Further paired *t*-tests were performed for aHipp and pHipp within the hemisphere for statistically significant FC (*p* < 0.05, FWE corrected).

*(2) FA Values*. Within-group analysis of the mean FA from the left and right hippocampi was conducted using one-sample *t*-test (*p* < 0.05, FWE corrected). Then, paired *t*-tests were performed for statistically significant hippocampus subregions (*p* < 0.05, FWE corrected).

#### 2.5.2. Age-Related Generalized Linear Model

Specifically, we created an age-related GLM to examine FC and FA changes. In order to investigate the reflections between brain regions and memory for ageing individuals, we established multiple linear regression equations with gender as covariate. *Y* represented the fitted value, *a*_0_ ~ *a*_3_ represented the regression coefficients of each factor, age and age^2^ were predictive factors, and the gender factor was a covariate. Concretely, the GLM model can be expressed with the following equations:

(1) $$\begin{array}{c} Y=a_{0}+a_{1}\times \text{age}+a_{2}\times \text{sex}, \end{array}$$

(2) $$\begin{array}{c} Y=a_{0}+a_{1}\times \text{age}+a_{2}\times {\text{age}}^{2}+a_{3}\times \text{sex}. \end{array}$$

The age-relevant prediction model was generated using Akaike's information criterion [5]. And the regression coefficients of model predictor variables were statistically analyzed using a one-sample *t*-test. For FC that exhibits significant quadratic age effects, the peak age could be calculated using the following formula:

(3) $$\begin{array}{c} {\text{Age}}_{\text{peak}}=\frac{-a_{1}}{a_{2}}. \end{array}$$

#### 2.5.3. Behavioral Scale Correlation Analysis

The Wechsler Abbreviated Scale of Intelligence (WASI) was a short form IQ test used for people from the ages of 6 to 89. The WASI consisted of four subtests, vocabulary (VC), similarities (SI), block design (BD), and matrix reasoning (MR) [39]. The scale was intended for retesting purposes and attaining quick estimates of cognitive ability. It was not intended as a substitute for a more comprehensive measure of intelligence or as a diagnostic instrument. To investigate the age-related relationship between cognitive ability and structural changes in hippocampus, we examined correlation between the FA values and WASI during lifespan. The WASI included verbal IQ (VERB: VC score + MR score + BD score) and vocabulary comprehension (VOCAB.T: VC score + SI score) (*p* < 0.05). Then, we plotted the fitted linear curve of the obtained FA values versus WASI scores and computed Pearson's correlation between the FA values across the range that centered on the peak age and behavioral scales (*p* < 0.05).

## 3. Results

### 3.1. Functional and Structural Statistical Results in Hippocampus

**Figure 1:**
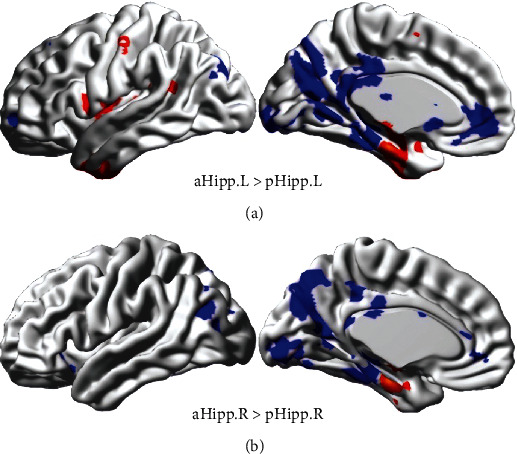
The FC different regions between aHipp and pHipp. (a) Statistical results in the left hemisphere. (b) Statistical results in the right hemisphere. The red parts indicated that the FC of aHipp was significantly greater than that of pHipp. The blue parts indicated that the FC of pHipp was significantly greater than that of aHipp (*p* < 0.05, FWE corrected).

In exploring hippocampal FC, a paired *t*-test was performed on the aHipp and pHipp after one-sample *t*-test in the left and right hemispheres, respectively. Some statistically significant FC brain regions are shown in Figure 1 (*p* < 0.05, FWE correction). Blue parts indicated that the FC of the pHipp was significantly greater than that of the aHipp. Red parts indicated that the FC of the aHipp was significantly greater than that of the pHipp. As we could see, the number of functional connections in pHipp was more than that in aHipp.  Figure 1(a) is the result of the left hemisphere, and details are shown in Table 1. In the left hemisphere, pallidum and inferior temporal gyrus had a significant FC with aHipp. Precuneus, ACC, calcarine, and lingual gyrus had a significant FC with pHipp.  Figure 1(b) is the result of the right hemisphere, and details are shown in Table 2. In the right hemisphere, only the fusiform gyrus had a significant FC with aHipp. Here, nine brain regions had significant FC with pHipp, such as anterior cingulate gyrus, inferior temporal gyrus, and superior occipital gyrus. More FC in the pHipp may indicate that it was involved in more information exchange processes and played a more important role.

**Figure 2:**
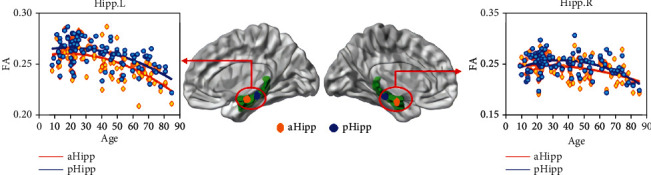
Trends of hippocampus white matter structural FA with age. The inverted U-shaped trajectories of FA included aHipp.L, pHipp.L, and pHipp.R. Orange plots represented the aHipp and blue plots the pHipp.

In exploring hippocampal FA, a paired *t*-test of FA values for aHipp and pHipp within the hemisphere (*p* < 0.05, FWE corrected) revealed that FA values of pHipp were significantly greater than FA values of aHipp (Figure 2). Here, the outstanding performance was also shown by pHipp.

### 3.2. Age-Related GLM Results

#### 3.2.1. Hippocampal FC across the Lifespan

**Figure 3:**
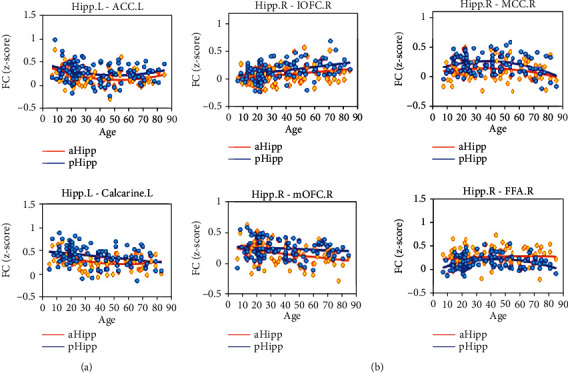
Trends of hippocampal functional connectivity with age. (a) The FC results of GLM in the left hemisphere. The U-shaped trajectories of FC included aHipp.L-ACC.L, pHipp.L-ACC.L, and aHipp.L-Calcarine.L. The linear downward trajectory of FC included pHipp.L-Calcarine.L. (b) The FC results of GLM in the right hemisphere. The inverted U-shaped trajectories of FC included pHipp.R-MCC.R and pHipp.R-FFA.R. The linear upward trajectories of FC included aHipp.R-lOFC.R and pHipp.R-lOFC.R. The linear downward trajectory of FC included pHipp.R-mOFC.R. Orange plots represented the aHipp and blue plots the pHipp (“.L”: “left hemisphere”; “.R”: “right hemisphere”).

We investigated the FC changes in hippocampus subregions from a lifespan perspective using an age-relevant GLM with linear and quadratic age terms as the predictive factor. The patterns that described the FC trend between hippocampus subregions and associated brain regions are shown in Figure 3. Four types of trends between FC and age were found in hippocampus subregions. In the left hemisphere (Figure 3(a)), the FC between aHipp.L and ACC.L had a significant quadratic age term (*t* = 2.85, *p* = 0.005, *R*^2^ = 0.16), and the FC between pHipp.L and ACC.L also had a significant quadratic age term (*t* = 1.95, *p* = 0.05, *R*^2^ = 0.06). They exhibited U-shaped trajectory with age. The FC between aHipp.L and Calcarine.L exhibited a significant quadratic age effect (*t* = 2.62, *p* = 0.01, *R*^2^ = 0.20) and demonstrated an age-related U-shaped trajectory. For the FC between pHipp.L and Calcarine.L, the linear term was found to be significant (*t* = −3.30, *p* = 0.001, *R*^2^ = −0.31), and the FC decreased linearly with age. In the right hemisphere (Figure 3(b)), the linear upward trend of FC with age was found to be significant in aHipp.R-lOFC.R (*t* = 3.08, *p* = 0.002, *R*^2^ = 0.29) and pHipp.R-lOFC.R (*t* = 4.21, *p* = 0.0001, *R*^2^ = 0.38). The FC between aHipp.R and mOFC.R decreased linearly as age increased (*t* = −3.28, *p* = 0.001, *R*^2^ = −0.31). The FC between pHipp.R and MCC.R exhibited a significant quadratic age effect (*t* = −2.85, *p* = 0.005, *R*^2^ = 0.11) and demonstrated an age-related inverted U-shaped trajectory. For the FC between pHipp.R and FFA.R, the quadratic age effect was also significant (*t* = −3.45, *p* = 0.0008, *R*^2^ = 0.11), and the FC exhibited an inverted U-shaped trajectory with age. We could see the U-shaped trajectory and the inverted U-shaped trajectory with the turning point of the change occurring around age 40. The specific correlation statistical parameters and fitting functions are shown in Table 3. Neither of the FC of pHipp.R-mOFC.R nor the FC of aHipp.R-MCC.R showed a significant correlation with age. GLM analysis revealed that the FC of aHipp.R-FFA.R was not statistically significantly correlated with age.

#### 3.2.2. Hippocampal FA across the Lifespan

For the hippocampal structure, we obtained specific patterns that described age-related changes in mean hippocampus subregion FA degree vs. age (Figure 2). Considering the wide age distribution (7-85 years), we tested nonlinear trends with a quadratic age function (see Equation (2) in Age-Related Generalized Linear Model). The FA exhibited increased trends during early ages and decreased trends at later ages, i.e., the inverted U-shaped trajectory with aging. The aHipp.L FA exhibited a significant quadratic age effect (*t* = −2.48, *p* = 0.01, *R*^2^ = 0.37) and demonstrated an age-related inverted U-shaped trajectory. For the pHipp.L FA, the quadratic term was significant (*t* = −2.42, *p* = 0.01, *R*^2^ = 0.34) and showed an IU trend with age. The pHipp.R FA presented a significant quadratic age effect (*t* = −3.78, *p* = 0.0003, *R*^2^ = 0.21) and demonstrated an age-related IU trend. The specific correlation statistical regression coefficients and quadratic fitting curve are shown in Table 4. GLM analysis revealed that the FA of aHipp.R was not statistically significantly correlated with age. Obviously, the FA value of pHipp was greater than aHipp throughout the life cycle.

#### 3.2.3. Correlation between FC and FA

**Figure 4:**
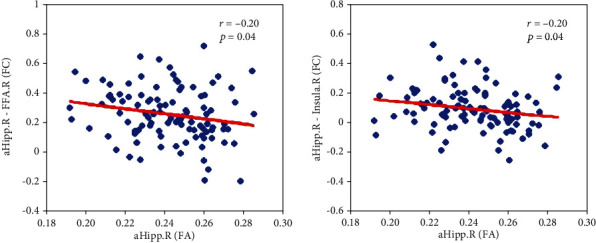
Correlation between FC values and white matter structural FA values in hippocampus subregions.

After calculating the FA and the FC between hippocampus subregions and other brain regions, we acquired the Pearson correlation between the FC and FA for hemispheric hippocampus subregions (Figure 4; *p* < 0.05, FDR corrected). The FC influence between aHipp.R and FFA.R was negatively correlated with the aHipp.R FA (*r* = −0.20, *p* = 0.04). The FC between aHipp.R and Insula.R was also negatively related with the aHipp.R FA (*r* = −0.20, *p* = 0.04). None of the other hemispheric hippocampus subregions exhibited significant correlations.

#### 3.2.4. Behavioral Scale Correlation Results

**Figure 5:**
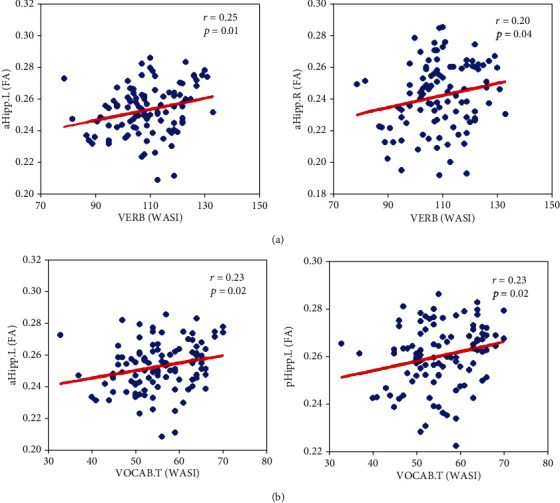
Correlation between hippocampal structural FA values and WASI scores. (a) Correlation between FA values and VERB scores. (b) Correlation between FA values and VOCAB.T scores.

For the entire lifespan, there was no statistically significant correlation between FC values and VERB scores and between FC values and VOCAB.T scores. However, there were significant correlations between the age-dependent linear fitting curve of FA and two WASI scales (VERB scores: 109.53 ± 11.37, VOCAB.T scores: 55.65 ± 7.71), as shown in Figure 5. Specifically, in Figure 5(a), the FA of aHipp.L was positively correlated with VERB scores (*r* = 0.25, *p* = 0.01), and the FA of aHipp.R was also positively correlated with VERB scores (*r* = 0.20, *p* = 0.04). Therefore, structural changes in the aHipp had an important impact on the performance of VERB. Furthermore, in Figure 5(b), the FA of aHipp.L was positively correlated with VOCAB.T scores (*r* = 0.23, *p* = 0.02), and the FA of pHipp.L was also positively correlated with VOCAB.T scores (*r* = 0.23, *p* = 0.02). Accordingly, structural changes in the left hippocampus were closely related to the performance of VOCAB.T.

## 4. Discussion

As a crucial constituent of the limbic system, the hippocampus represents an anatomical and functional substrate that synthesizes information from multiple memories. Our study is conducted in 106 subjects aged 7-81 years. We acquire lifetime patterns which describe functional and structural changes in distinct hippocampus subregions based on an age-related GLM.

In particular, the data provide four main results. (1) Compared with the FC of aHipp, the FC of pHipp is stronger, especially in the pHipp.R. (2) In terms of the white matter structure of hippocampus, the FA value of pHipp is larger than that of aHipp. (3) In terms of the structural-functional coupling in hippocampus, we observe a significant negative correlation between FA value of aHipp.R and FC value of aHipp.R-FFA.R and a significant negative correlation between FA value of aHipp.R and FC value of aHipp.R-insula. In other words, there is no obvious coupling between the functions and structures of other parts except these two parts. (4) In terms of the influence of age, by using GLM, we find that the aHipp and pHipp show age-related FC changes with different trends, and the difference peaks at about 40 years old. At the same time, only FA of aHipp.R is not significantly affected by age. Combined with WASI evaluation results, FA changes in hippocampus white matter structure affect the verbal IQ and vocabulary comprehension ability of the subjects significantly.

Overall, these results provide strong evidence that age-related memory changes are closely related to the hippocampus region. Namely, functional separation and integration of the two regions (aHipp and pHipp), as well as changes in white matter structure, are modulated by age. The implications of these main findings are discussed in the following.

### 4.1. Functional Connectivity in the Hippocampus

Here, it was found that aHipp and pHipp had significant FC with different brain regions, respectively. Three brain regions (pallidum, inferior temporal gyrus, and FFA) were associated with aHipp significantly, while 13 brain regions (precuneus, cingulate gyrus, lingual gyrus, orbitofrontal cortex, and so on) were associated with pHipp. The role of the hippocampus in memory depended on its interaction with distributed brain regions. The regions associated with aHipp have prominent contributions in regulating working memory [1], recognizing memory objects [40], and perceiving faces [41]. Their FC with aHipp was an important indicator of early memory formation and consolidation [42]. In addition, the regions associated with pHipp have prominent contributions in perceiving directional memory [43], situational memory and spatial processing [44], preserving memory [45], manipulating and monitoring [46], and extracting information [47]. These brain regions are involved to a greater extent in memory retention and retrieval. Also, many hippocampal studies have reported that aHipp was involved in encoding in memory [48, 49], and pHipp was more concerned with repetitive stimulus and memory retrieval [7, 50]. Different brain regions may be involved in different memory types (e.g., working memory and long-time memory) [51, 52], so the collaboration of the hippocampus with these brain regions contributed to adapting the integrative processing of memory information [53, 54]. The strength of FC in the aHipp and pHipp varied greatly and involved different brain regions, which supported the functional separation between the aHipp and pHipp. Namely, aHipp may preferentially support novel stimulus and memory encoding, while pHipp may preferentially support repetitive stimulus and memory retrieval. Their FC to the same brain regions may be the reflection of functional integration. From the perspective of FC, our results further demonstrated the existence of functional separation and integration of aHipp and pHipp during growth and aging.

Our results showed dominant functional connectivity in the pHipp. It was consistent with previous work by Blum et al., which revealed a shift from aHipp dominant hippocampus connectivity in younger age group to pHipp dominant connectivity in aging subject, suggesting an age-related reorganization of hippocampal networks supporting normal cognitive function [17]. Other studies have also reported that aHipp atrophied before pHipp during aging [55, 56]. Thus, the FC of aHipp may be diminished with age, and those in pHipp may be compensated for by increased connectivity with neocortical regions in the elderly.

We also found that the hippocampal FC showed U-shaped, inverted U-shaped, linear upward, and linear downward trends with age. The FC of aHipp.L-ACC.L and aHipp.L-Calcarine.L showed a U-shaped trajectory (FC decreased during early growth and maturation but increased during late aging), which may be related to the development of brain function. Previous studies have shown that ACC, calcarine, and aHipp were important regions associated with working memory [43, 48], and ACC was involved in remote memory retrieval [57]. Combined with relevant studies, the high FC in childhood may signify less developed subregional specialization of communication, less neural processing efficiency, or lack of inhibition [14]. In the growth process, due to the improvement of neural processing efficiency and the accumulation of long-term memory, there was no need to stimulate more connections in the hippocampus. Thus, aHipp was uncoupled with ACC and calcarine [57]. The high FC in the elderly may be cognitive compensation for overrecruitment in activity [58, 59]. If overrecruitment in aging existed for a given task as a result of cognitive compensation, then one could envision that also FC would be higher. This trend conformed to the standard model of memory system consolidation. As memory function matures, its storage and retrieval became increasingly independent of the hippocampus, and in the aging process, memory consolidation would be more dependent on the hippocampus [13]. Other research results also supported the U-shaped trajectory of the brain regions related to working memory in this study and provided a neural basis explanation [60].

The FC of aHipp.R-lOFC.R and pHipp.R-lOFC.R showed a linear upward trajectory. With the increase of age, the FC between lOFC and hippocampus would be closer. Pudas et al.'s research showed that individuals with impaired memory have increased FC in the frontal lobe during memory encoding and retrieval [61]. lOFC had the ability to coordinate and maintain memory. So, the increase of functional coupling between lOFC and hippocampus may be related to the increasing amount of memory information and the synergy of memory processing. Interestingly, hippocampus FC appeared to increase in late ages, which also seemed to occur with the expression of some hippocampal cell markers. On the one hand, it has been shown that brain aging involved regional alterations of specific cellular subpopulations in the human hippocampus [62, 63]. For example, increased glutamatergic transporter was observed in multiple hippocampal subfields at late ages. This glutamatergic marker was positively correlated with beta-amyloid and tau proteins [63]. The GA1 hippocampal area was also positively correlated with the concentrations of interferon-gamma and brain-derived neurotrophic factor (BDNF). It was also relevant to note that BDNF played a central role in neurophysiological plasticity processes, such as memory and learning, which depend on the hippocampus function [64]. On the other hand, West's work showed that the total number of some brain neurons involved in memory processes declined with age, but hippocampus subregions showed no significant change [62]. The losses qualified as potential morphological correlates of senescent decline in relational memory, because they could be expected to compromise the functional integrity of a region of the brain known to be intimately involved in this type of memory. So, the increases in hippocampal FC in later life might be a compensation for these losses.

The FC between pHipp.R-FFA.R and pHipp.R-MCC.R showed an inverted U-shaped trajectory. Namely, FC in the youth was higher than FC in children and the elderly. It suggested that the pHipp functional coupling of the facial perception and the memory integration was enhanced during development but weakened during aging. This inverted U-shaped trajectory was also reflected in study of hippocampal memory from Abrari et al. and Chowdhury et al. [65, 66]. Age-related FC declines included mOFC and calcarine. They were associated with maintaining memory. This synchronous decline may contribute to better maintenance of memory cognition with hippocampus atrophy [67]. The differences in FC strength between the anterior and posterior hippocampi and the same brain regions reached their maximum or minimum at about age 40, which may represent that 40 was a sign of changes in brain function and the starting point of aging phenomenon. This phenomenon also answered a key question of neurology and knew when brain functions stopped to mature and when they started to degenerate [68]. All in all, these trends provided an effective insight into the functional separation and integration of brain regions related to memory throughout the life cycle and laid a theoretical foundation for understanding the neural mechanism of memory changes.

### 4.2. Structural Change in the Hippocampus

Memory performance was also highly dependent on structural changes in the hippocampus. Structurally, our study found that the FA value of white matter in pHipp was significantly higher than that of aHipp, indicating that the integrity of pHipp white matter was higher than that of aHipp white matter. This result provided additional infrastructural information for the functional specificity of the aHipp and pHipp. Consistent with the results of FC, the pHipp highlighted its importance throughout the life cycle. Previous research has shown that individuals with high FA in the hippocampus were faster in forming a cognitive map of the environment and more effective in spatial orientation [69]. According to the study of Hickie, the hippocampus was mainly responsible for memory and emotional control, and if this part shrank, the corresponding ability of memory and emotional control was weakened [70]. At the same time, studies have confirmed the hypothesis about the functional specialization of the anterior and posterior hippocampi: the aHipp was strongly associated with memory encoding and the pHipp with memory retrieval [7]. And prior DTI studies have typically found associations between higher FA and better memory performance [71, 72]. Combined with the results of previous studies, our results showed that the FA in pHipp was greater than that in aHipp, which may represent a stronger involvement of the pHipp in memory scenes throughout the life cycle. Meanwhile, this may mean that the brain received more repetitive stimuli than novel ones and processed them more efficiently throughout our life cycle. The frequency of memory retrieval and extraction may be higher than that of memory encoding.

However, in terms of behavior-related outcomes, more effects were observed in aHipp regions. The influencing factors came from the functional properties of the aHipp and pHipp on the one hand, and the properties of the cognitive scales on the other. The VERB scale measured cognitive ability in terms of verbal intelligence, which depended more on the individual's learning ability. And the VOCAB.T scale measured cognitive ability in terms of vocabulary comprehension, which depended more on the experience gained from prior learning. Compared to the process of retrieving memories, the brain dispatched more resources when encoding new memories, and both learning ability and prior experience influenced encoding efficiency. In general, aHipp was the primary input brain region associated with encoding new memories, while pHipp was the output brain region associated with memory retrieval and consolidation [3]. Therefore, in the scale correlation analysis of the present study, aHipp showed a more significant association with behavior.

Further, our results supported the functional specificity of the aHipp and pHipp and the functional segregation that developed with age. In terms of trajectory, we found that with the increase of age, the change of FA showed an inverted U-shaped trajectory, and the peak occurs around the age of 30. This was consistent with previous studies [73, 74], where Coupé et al. found that white matter trajectory based on absolute and normalized volumes followed an inverted U-shape with a maturation peak around midlife [73]. These results indicated that during the early phase of brain development, white matter expansion exceeded general growth, but there was a contraction of hippocampal white matter from maturity to aging, which may contribute to age-related memory decline [75]. On a microscopic level, aHipp was mainly connected with uncinate fasciculus, and pHipp was mainly connected with limbic association bundle. Hasan et al. found that the FA value of the unhooked fascicles follows an inverted U-shaped trajectory with age throughout the life cycle [76]. Memory and FA of cingulate bands were significantly positively correlated during growth [77], while FA tended to decline during aging [78]. Therefore, the increase of FA during development and the decrease of FA during aging provided early predictors for the prevention and diagnosis of memory-related neural diseases. The above results supported the trend of inverted U-shaped FA in the white matter throughout the life cycle, which helped us to understand the neurophysiological basis of the decline in natural memory.

### 4.3. Coupling between Functional Connectivity and Structural Change

When exploring the structural-functional coupling in hippocampus, we found a significant negative correlation between FA of aHipp.R and FC of aHipp.R-FFA.R and a significant negative correlation between FA of aHipp.R and FC of aHipp.R-insula. In other words, when the FA of aHipp.R white matter increased, the significantly correlated FC decreased. Previous studies on temporal lobe epilepsy showed that the structural-functional decoupling was mainly attributable to structural and functional changes in the hippocampus, frontal inferior orbital gyrus, and posterior cingulate, and the right structural-functional decoupling involved predominantly bilateral hippocampal functional changes [79]. Presumably, the negative correlation between FA and FC in hippocampus may reflect either a compensatory process. The increase of FA in aHipp.R during the memory change to compensate for the decoupling is caused by FC loss. A study of patients with major depressive disorder supported our results. They identified a negative correlation between uncinate fasciculus's white matter integrity and subgenus ACC's FC with the bilateral hippocampus [80]. Namely, structural abnormalities of patients contributed to the increase of FC in the frontal neural network. However, only FC of aHipp.R was associated with white matter structural integrity, while the pHipp's FC was not. Therefore, to some extent, this result suggested that more are internally coupled between structure and function in aHipp, and the development of function was constrained by structure during human lifetime.

In the analysis of WASI, our results showed that FA of aHipp.L was positively correlated with VERB and VOCAB.T. FA of aHipp.R was only positively correlated with VERB. FA of pHipp.L was only positively correlated with VOCAB.T. These positive correlations suggested that hippocampal white matter structural integrity was strongly linked to intelligence and comprehension in healthy individuals, but FC was not affected by these capabilities. Previous studies have shown that word comprehension was associated with long-term memory [81], and verbal IQ was associated with white matter fiber damage [82]. We postulated that the hippocampus directly or indirectly affected memory-related cognitive abilities through white matter fibers, and a reduction in hippocampal volume was associated with impairment of general knowledge and contextual visual memory [83]. Fjalldal et al. reported that changes in white matter integrity in the right hippocampus were associated with decreased visual spatial capacity, whereas decreased white matter integrity and demyelination/edema in the left hippocampus were associated with impaired general knowledge and delayed recall in episodic memory [84]. Therefore, this provided a new way for us to understand the life-cycle mechanism of memory change, that is, the relationship between the structural integrity of hippocampal white matter and cognitive ability. Meanwhile, the structural-functional coupling of the hippocampus allowed us to assess age-related differences in cognitive ability, which may serve as a potentially sensitive marker for improving memory across the lifespan.

### 4.4. Limitations

This study has several limitations. Firstly, we used a relatively small sample size across the human lifespan, which may have prevented the detection of biological associations. Nevertheless, we were still able to reveal significant differences. Studying a larger sample size will likely allow further details to be uncovered. Secondly, it must also be noted that although the focus of the study was hippocampal changes across the lifespan, we did not sample children below 7 years, but performance during infant and early childhood development was also significantly important. This was a limitation, as this period was likely the most important in development of episodic memory function. Thirdly, we employed linear and quadratic (nonlinear) models to explore the hippocampal FC and FA changes across the human lifespan. The incomplete distribution of ages in our sample may have affected parametric curve fitting. In future studies, exploration of larger fMRI and DTI datasets using nonparametrical models (e.g., smoothing splines) may reveal more robust and complex maturational processes [85].

Research on the hippocampus still limited in preventing the progression of memory-related dementia. The most valuable finding in our study was that age 40 was the beginning of the decline in the memory capacity (trajectory of FC and FA with age). Therefore, age 40 was the prime time to intervene and prevent the occurrence of memory diseases. After age 40 was also accompanied by a decline in the structural integrity of hippocampus subregions, a phenomenon was also an important and useful indicator of cognitive decline and dementia development.

## 5. Conclusions

In this paper, the functional and structural characteristics of hippocampus across the lifespan were studied by resting fMRI and DTI. The functional integration and separation between aHipp and pHipp were explored, and findings supported more functional separation and less functional integration between the aHipp and pHipp. At the same time, the U-shaped trajectory, linear upward trajectory, inverted U-shaped trajectory, and linear decline trajectory of FC changes between hippocampus and memory-related brain regions throughout the life cycle were discussed in detail. The structural integrity of aHipp.L, aHipp.R, pHipp.L, and pHipp.R across the lifespan was explored. Our study provided new evidence for the functional integration, functional separation, and the structural integrity of aHipp and pHipp with age. The structural and functional changes of aHipp and pHipp across the lifespan may be a reliable biomarker in clinical used to provide a theoretical basis for the pathophysiology of different neuropsychiatric disorders (e.g., dementia, Alzheimer's, and schizophrenia).

## Figures and Tables

**Table 1:** The final significant FC regions of aHipp.L and pHipp.L within the left hemisphere.

ROI	Anatomical	Hemisphere	Cluster voxels	MNI (*x*, *y*, *z*)	*T*-value
aHipp	Pallidum	L	23	(-21, 0, -6)	7.6
aHipp	Inferior temporal gyrus	L	15	(-33, 12, -39)	6.62
pHipp	Precuneus	L	28	(-9, -69, 36)	-5.04
pHipp	Anterior cingulate cortex	L	20	(-12, 39, -6)	-5.04
pHipp	Calcarine cortex	L	46	(-6, -63, 18)	-5.06
pHipp	Lingual gyrus	L	17	(-15, -93, -12)	-5.06

**Table 2:** The final significant FC regions of aHipp.R and pHipp.R within the right hemisphere.

ROI	Anatomical	Hemisphere	Cluster voxels	MNI (*x*, *y*, *z*)	*T*-value
aHipp	Fusiform gyrus	R	34	(39, -18, -33)	5.29
pHipp	Anterior cingulate cortex	R	16	(9, 33, 18)	-5.06
pHipp	Inferior temporal gyrus	R	20	(51, -51, -12)	-5.07
pHipp	Superior occipital gyrus	R	15	(18, -87, 27)	-5.07
pHipp	Insula	R	21	(27, 24, -12)	-5.10
pHipp	Lateral orbital frontal cortex	R	16	(33, 27, -6)	-5.10
pHipp	Medial orbital frontal cortex	R	17	(9, 51, -6)	-5.22
pHipp	Precuneus	R	530	(21, -54, 15)	-9.18
pHipp	Lingual gyrus	R	98	(9, -87, -3)	-6.73
pHipp	Middle cingulate cortex	R	61	(9, -33, 36)	-6.75

**Table 3:** Results of multiple regression analyses between FC and age.

FC	*T*	*p*	*R* ^2^/**R**^2^	Fitting curve
Left hemisphere				
aHipp.L-ACC.L	2.85	0.005	0.16	*y* = 0.00011age^2^ − 0.01220age + 0.43617
aHipp.L-Calcarine.L	2.62	0.01	0.20	*y* = 9.7516*e*^−05^age^2^ − 0.01159age + 0.47473
pHipp.L-ACC.L	1.95	0.05	0.06	*y* = 9.8348*e*^−05^age^2^ − 0.01024age + 0.47208
pHipp.L-Calcarine.L	-3.30	0.001	-**0.31**	*y* = −0.00302age + 0.42229
Right hemisphere				
aHipp.R-lOFC.R	3.08	0.002	**0.29**	*y* = 0.00214age − 0.00825
aHipp.R-mOFC.R	-3.28	0.001	-**0.31**	*y* = −0.00247age + 0.26402
pHipp.R-FFA.R	-3.45	0.0008	0.11	*y* = −0.00012age^2^ + 0.010969age − 0.031411
pHipp.R-lOFC.R	4.21	<0.0001	**0.38**	*y* = 0.00302age + 0.03234
pHipp.R-MCC.R	-2.85	0.005	0.11	*y* = −0.00011age^2^ + 0.00827age + 0.10145

Note: the bold **R**^2^: linear relationship (age); the light *R*^2^: nonlinear relationships (age^2^).

**Table 4:** Results of multiple regression analyses between FA and age.

FA	*T*	*p*	*R* ^2^	Fitting curve
Left hemisphere				
aHipp.L	-2.48	0.01	0.37	*y* = −8.1174*e*^−06^age^2^ + 0.00029age + 0.25736
pHipp.L	-2.42	0.01	0.34	*y* = −6.988*e*^−06^age^2^ + 0.00027age + 0.26303
Right hemisphere				
pHipp.R	-3.78	0.0003	0.21	*y* = −1.8869*e*^−05^age^2^ + 0.00135age + 0.23287

## Data Availability

Publicly available datasets were analyzed in this study. This data can be found here: http://fcon_1000.projects.nitrc.org/indi/pro/nki.html.
